# Diagnostic Accuracy of Hepatic Subcapsular Flow on Color Doppler Ultrasonography and Preoperative Liver Biopsy for the Diagnosis of Biliary Atresia in Neonatal Cholestasis: A Pilot Diagnostic Accuracy Study

**DOI:** 10.7759/cureus.110877

**Published:** 2026-06-15

**Authors:** Santosh K Mahalik, Sandhya Biswal, Suvradeep Mitra, Aditya A Manekar, Akash B Pati, Bikasha B Tripathy, Manoj K Mohanty

**Affiliations:** 1 Pediatric Surgery, All India Institute of Medical Sciences, Bhubaneswar, Bhubaneswar, IND; 2 Pathology and Laboratory Medicine, Kalinga Institute of Medical Sciences, Bhubaneswar, IND; 3 Histopathology, Postgraduate Institute of Medical Education and Research (PGIMER), Chandigarh, IND

**Keywords:** biliary atresia, color doppler ultrasonography, hepatic subcapsular flow, hepatobiliary scintigraphy, liver biopsy, neonatal cholestasis, per-operative cholangiogram

## Abstract

Background

Biliary atresia (BA) is a progressive fibro-inflammatory obliterative cholangiopathy that is the most common surgical cause of neonatal cholestasis (NC) and the leading indication for pediatric liver transplantation. Early and accurate diagnosis is critical, as outcomes of Kasai hepatic portoenterostomy are significantly better when performed within 60 days of life. Hepatic subcapsular flow (HSF) on color Doppler ultrasonography (USG) has been proposed as a non-invasive diagnostic marker for BA, but its performance has not been adequately validated in diverse clinical settings.

Objectives

This study aimed to evaluate the diagnostic accuracy of HSF on color Doppler USG as the primary index test and percutaneous liver biopsy and hepatobiliary scintigraphy as comparator index tests for the diagnosis of BA in infants with NC, using per-operative cholangiogram (POC) as the reference standard.

Methods

This was a pilot cross-sectional diagnostic accuracy study conducted at a single tertiary care center between August 2018 and July 2021, reported in accordance with the Standards for Reporting Diagnostic Accuracy Studies (STARD) 2015 guidelines. Infants up to six months of age with conjugated hyperbilirubinemia were enrolled consecutively. All participants underwent fasting abdominal USG with color Doppler assessment of HSF, hepatobiliary scintigraphy (hepatobiliary iminodiacetic acid (HIDA) scan), and USG-guided percutaneous liver biopsy (evaluated using the Chen scoring system, threshold ≥8). The reference standard was POC. Sensitivity, specificity, positive predictive value (PPV), negative predictive value (NPV), and overall diagnostic accuracy were calculated for each index test against POC.

Results

Thirty-four patients were enrolled (22 males, 12 females; mean age 3.09 ± 1.46 months). BA was confirmed in 25 (73.5%) patients on POC. HSF was detected in eight patients (23.5%), all of whom had confirmed BA: sensitivity 32.0%, specificity 100%, PPV 100%, NPV 34.6%, and overall accuracy 50.0%. HIDA scintigraphy demonstrated sensitivity of 100%, specificity of 22.2%, PPV of 78.1%, NPV of 100%, and overall accuracy of 79.4%. Liver biopsy (Chen score ≥8): sensitivity 100%, specificity 22.2%, PPV 78.1%, NPV 100%, and accuracy 79.4%. The mean Chen score was 9.08 in BA versus 8.78 in non-BA patients (p = 0.207). Liver biopsy suspicion of BA correlated significantly with the POC reference standard (p = 0.014).

Conclusion

HSF on color Doppler USG demonstrated high specificity but low sensitivity for the diagnosis of BA in this cohort, limiting its utility as a stand-alone screening tool. Liver biopsy demonstrated the most clinically informative overall diagnostic performance. With the exception of HSF sensitivity, all reported diagnostic accuracy estimates in this pilot study should be regarded as exploratory, given limited statistical power. These findings should be interpreted cautiously because of the small sample size, late presentation profile, and operator variability. Larger prospective multicenter studies are required to validate the diagnostic utility of HSF.

## Introduction

Neonatal cholestasis (NC) is defined as conjugated hyperbilirubinemia occurring in the newborn as a consequence of impaired bile formation or flow. It is a potentially life-threatening condition with an incidence of approximately 1 in 2,500 live births in Western countries, and constitutes 19%-33% of all chronic liver diseases in children presenting to tertiary care centers in India [1,2]. Among obstructive causes, biliary atresia (BA) accounts for approximately 34% of cases and is the most common indication for pediatric liver transplantation worldwide [2].

BA is a progressive fibro-inflammatory obliterative cholangiopathy of the extrahepatic and intrahepatic biliary ducts. The Kasai hepatic portoenterostomy remains the primary surgical intervention and demonstrates substantially improved biliary drainage rates and long-term native liver survival when performed within 60 days of life; outcomes deteriorate markedly when surgery is delayed beyond 90 days [3]. Establishing an accurate preoperative diagnosis is therefore both time-sensitive and clinically critical.

The diagnostic workup for BA currently relies on a combination of clinical assessment, biochemical investigations (including serum gamma-glutamyl transpeptidase (GGT)), fasting abdominal ultrasonography (USG), hepatobiliary scintigraphy (hepatobiliary iminodiacetic acid (HIDA) scan), and percutaneous liver biopsy, with per-operative cholangiogram (POC) serving as the definitive reference standard. Each modality has well-recognized limitations in isolation [1,4]. Hepatobiliary scintigraphy has a pooled specificity of only 68.5%-72.2% and cannot reliably distinguish biliary obstruction from severe hepatocellular dysfunction [1,5]. USG has an overall diagnostic accuracy of approximately 75% for BA, with the triangular cord sign and gallbladder morphological abnormalities being the most diagnostically valuable parameters [1,4,6].

Hepatic subcapsular flow (HSF) on color Doppler USG has been proposed as a potentially high-yield, non-invasive marker for BA. Lee et al. reported 100% sensitivity and 86% specificity, and El-Guindi et al. reported 96.3% sensitivity and specificity in prospective cohorts [7,8]. The pathophysiological basis involves hyperplastic and hypertrophic arterial changes in the subcapsular hepatic parenchyma driven by progressive biliary fibrosis and cirrhosis [9]. However, HSF assessment is highly operator-dependent, lacks standardized threshold values in the pediatric population, and has not been validated in large or diverse patient cohorts. Its role in routine diagnostic algorithms remains undefined.

This study was therefore designed as a cross-sectional diagnostic accuracy study to evaluate whether HSF on color Doppler USG can serve as a reliable non-invasive diagnostic marker for BA in infants with NC. Using POC as the reference standard, the diagnostic performance of HSF was compared with that of liver biopsy and hepatobiliary scintigraphy, which were evaluated as established comparator index tests within the current diagnostic workup for BA.

## Materials and methods

Study design

This was a cross-sectional diagnostic accuracy study conducted at a single tertiary care academic pediatric surgical center, reported in accordance with the Standards for Reporting Diagnostic Accuracy Studies (STARD) 2015 guidelines [10]. Ethical approval was obtained from the Institutional Ethics Committee (IEC reference number: T/IM-NF/Ped.Surg/17/33). Written informed consent was obtained from the parent or legal guardian of every enrolled patient prior to any study procedures.

Study period and setting

Patient enrollment was carried out from August 2018 to July 2021 at a tertiary care center. Recruitment was adversely affected in 2020 due to the COVID-19 pandemic and associated institutional shutdowns, resulting in a limited sample size.

Participants

Infants aged two weeks to six months presenting with jaundice persisting beyond two weeks were consecutively screened for NC. Inclusion required confirmed conjugated hyperbilirubinemia, defined as direct bilirubin greater than 1.0 mg/dL when total serum bilirubin was less than 5.0 mg/dL, or greater than 20% of total serum bilirubin when total serum bilirubin was 5.0 mg/dL or higher, along with parental consent. Infants with a previously established metabolic, genetic, or infectious cause of cholestasis or without consent were excluded.

Sample size considerations

No formal a priori sample size calculation was performed, as this was a pilot diagnostic accuracy study and the precise prevalence of HSF in the target population was unknown at the time of study design. Patient enrollment was intended to be consecutive over the three-year study period. Recruitment was curtailed by the COVID-19 pandemic, resulting in a final sample of 34 patients. Post hoc, using a reported sensitivity of HSF of approximately 96% [8] and a 95% confidence interval half-width of 20%, a minimum sample of approximately 15 BA-positive cases would be required; our cohort of 25 BA cases satisfies this criterion for sensitivity estimation, though the confidence intervals around specificity and accuracy remain wide owing to the small non-BA subgroup (n = 9). These limitations are explicitly acknowledged, and the findings should be regarded as preliminary and hypothesis-generating.

This pilot study was not powered to estimate specificity for any of the three index tests with adequate precision, nor was it powered to detect the observed difference in mean Chen scores between BA and non-BA groups (9.08 vs 8.78), which would require a substantially larger sample given the small effect size and overlapping distributions. The study was designed and is presented as hypothesis-generating with respect to all metrics other than HSF sensitivity, for which the achieved sample of 25 BA-positive cases meets the minimum threshold calculated post-hoc.

Index tests

Three index tests were evaluated against the reference standard (POC). HSF on color Doppler USG was the primary index test. HIDA scintigraphy and percutaneous liver biopsy were evaluated as comparator index tests to contextualize the performance of HSF within the existing standard diagnostic workup. The intent of all three tests in this study was diagnostic (i.e., to determine the presence or absence of BA at the time of evaluation), not purely screening. The three tests were applied independently, and interpreters were not informed of the results of other index tests prior to their assessments.

Index Test 1: USG With Color Doppler Assessment of HSF

Fasting color Doppler USG was performed using a high-frequency linear transducer (7.5-12 MHz) with standardized Doppler settings (pulse repetition frequency 0.6-1.0 kHz; low-velocity filter). Parameters assessed included liver size and echotexture, gallbladder size and morphology (including post-prandial contractility), visualization of the common bile duct, and the triangular cord sign at the porta hepatis. HSF was defined as arterial flow signals detectable in the subcapsular hepatic parenchyma at a depth of less than 1 cm from the liver capsule, distinct from the main intrahepatic vasculature, on color Doppler imaging. HSF was recorded as present or absent. Assessments were performed by consultant radiologists with a minimum of three years of post-fellowship experience in pediatric hepatobiliary USG. It is acknowledged that HSF assessment was performed by more than one radiologist across the study period in the absence of a formal standardized acquisition protocol and that no validated quantitative thresholds for abnormal HSF exist for the pediatric population; these are explicitly recognized as inherent limitations of this index test and as factors likely contributing to the reduced sensitivity observed.

Index Test 2: Hepatobiliary Scintigraphy (HIDA Scan)

Technetium-99m-labeled hepatobiliary imiodiacetic acid (^99mTc-HIDA) scintigraphy was performed after phenobarbitone pretreatment (5 mg/kg/day orally for five days prior to the scan). The administered radiotracer dose was calculated at 1.85 MBq/kg (0.05 mCi/kg), with a minimum dose of 18.5 MBq (0.5 mCi) in accordance with standard pediatric nuclear medicine dosimetry guidelines. Anterior planar images were acquired at five-minute intervals for 60 minutes, followed by a delayed image at 24 hours. Interpretation was performed by a nuclear medicine physician blinded to the results of the other index tests. Failure to demonstrate tracer excretion into the intestinal lumen by 24 hours was defined as a non-excretion/obstructive pattern and classified as a positive result for BA; demonstration of tracer activity in the bowel at any time point was classified as preserved excretion and a negative result for BA.

Index Test 3: Percutaneous Liver Biopsy

USG-guided percutaneous needle liver biopsy was performed in all enrolled patients after correction of coagulopathy, where required. All biopsy specimens were evaluated independently by two experienced pathologists, both of whom were blinded to the results of the other index tests (HSF USG and HIDA scintigraphy) at the time of histopathological reporting. The Chen scoring system was applied as described in the original publication [11], with component scores assigned for bile duct proliferation, portal fibrosis, bile plug formation, and lobular changes. In cases of discordance between the two pathologists, consensus was reached by joint review. The dual independent reporting design strengthens the internal validity of the histopathological assessments; however, formal inter-observer agreement statistics (e.g., Cohen's kappa) were not pre-specified and were not calculated, which we acknowledge as a limitation. We recommend that future multicenter studies incorporate prospective inter-rater reliability assessment as a protocol-defined endpoint.

Reference standard

The reference standard for the definitive diagnosis of BA was POC, performed at laparotomy by an experienced consultant pediatric surgeon. Failure to demonstrate a patent biliary system on cholangiography confirmed BA, following which Kasai hepatic portoenterostomy was performed in the same operative setting. In the two patients in whom hepatobiliary scintigraphy convincingly demonstrated tracer excretion into the intestine, the diagnosis of non-BA was established, and POC was not performed (these patients were classified as non-BA).

Post-operative management

Patients with confirmed BA who underwent Kasai portoenterostomy were managed post-operatively with ursodeoxycholic acid, prophylactic antibiotics, and fat-soluble vitamin supplementation (A, D, E, and K). Follow-up was conducted at weekly intervals in the outpatient department to assess for jaundice clearance (defined as total serum bilirubin <2.0 mg/dL), change in stool color, and development of complications, including cholangitis and ascites.

Outcome measures and statistical analysis

Patients were classified as BA or non-BA based on the POC reference standard. For each index test, a 2 × 2 contingency table was constructed against the POC result to derive: sensitivity, specificity, positive predictive value (PPV), negative predictive value (NPV), and overall diagnostic accuracy, with corresponding 95% confidence intervals where calculable given the sample size.

Continuous variables are expressed as mean ± standard deviation (SD). Categorical variables are expressed as frequency and percentage. Between-group comparisons of continuous variables were performed using the independent samples t-test. Associations between categorical variables were assessed using Fisher's exact test or the chi-square test as appropriate. A p-value of <0.05 was considered statistically significant. All analyses were performed using IBM SPSS Statistics for Windows, Version 26 (Released 2018; IBM Corp., Armonk, NY, USA).

## Results

**Figure 1:**
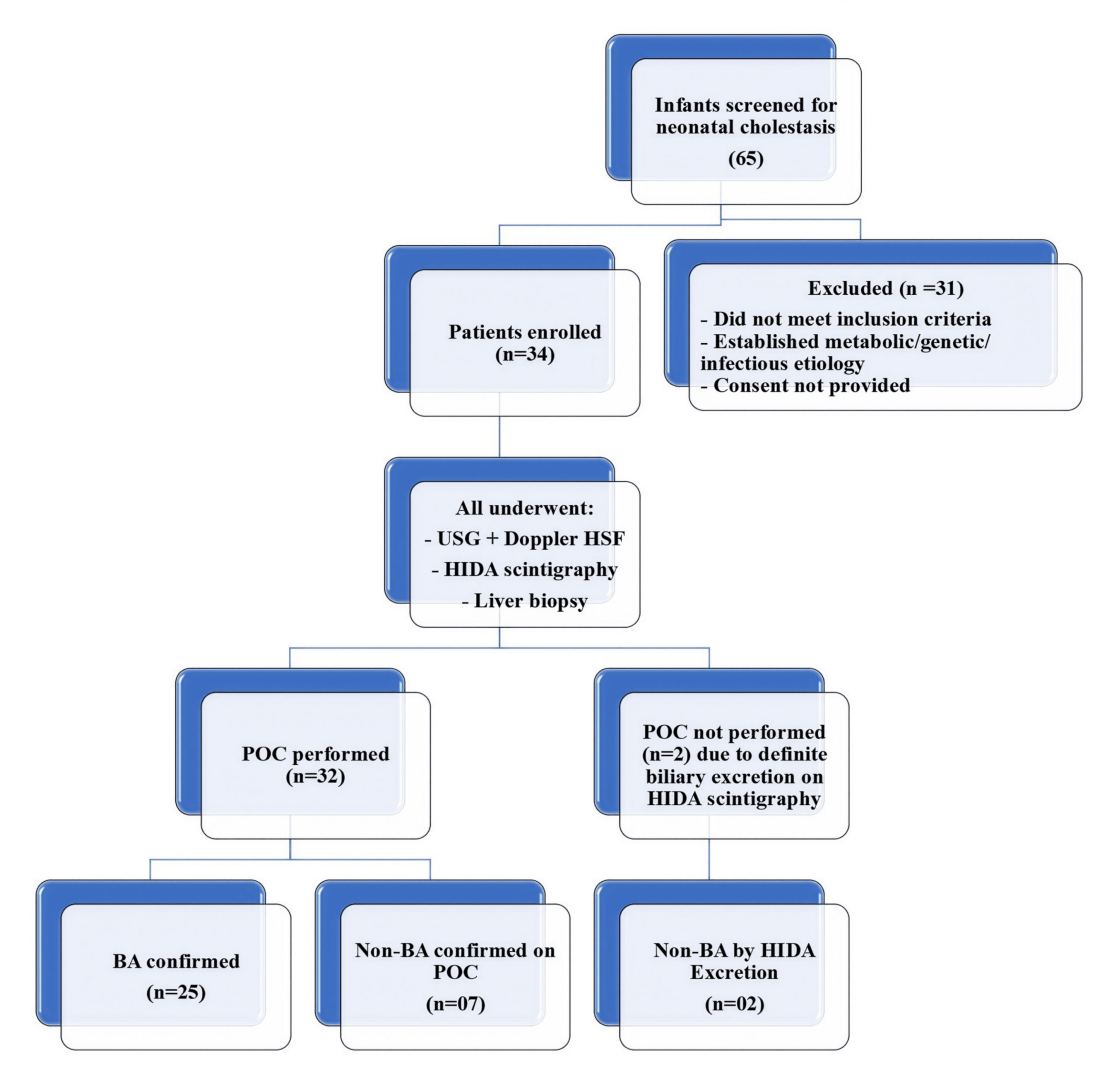
STARD flow diagram of study enrollment and diagnostic evaluation. STARD 2015-compliant flow diagram showing patient screening, enrollment, application of index tests, use of POC as the reference standard, and final diagnostic classification. BA: biliary atresia; POC: per-operative cholangiogram; USG: ultrasonography; HIDA: hepatobiliary iminodiacetic acid; HSF: hepatic subcapsular flow

The study enrollment process and application of index tests and reference standards are summarized in the STARD flow diagram (Figure 1). 

Patient demographics and clinical characteristics

Thirty-four patients were enrolled during the study period. Of these, 22 (64.7%) were male and 12 (35.3%) were female (male-to-female ratio 1.8:1). The mean age at presentation was 3.09 ± 1.46 months (range 1-6 months). Eight patients (23.5%) were born preterm. The late mean age at presentation (3.09 months) is an important contextual factor for interpreting the diagnostic accuracy findings, as it is beyond the optimal surgical window of 60 days and reflects the referral patterns of the study setting.

All 34 patients (100%) presented with jaundice, with onset documented as early as 10 days of life. Pale stools and dark urine were universally present. Abdominal distension was reported in 78% of patients. Hepatomegaly was detected in 33 patients (97.1%) and ascites in six (17.7%). Associated congenital anomalies were identified in a subset: umbilical hernia (6, 17.7%), proximal penile hypospadias (2, 5.9%), inguinal hernia (1, 2.9%), and bilateral pelvi-ureteric junction obstruction (1, 2.9%). Fundoscopy was normal in 31 patients (91.2%); bilateral central retinal atrophy was noted in three (8.8%).

Reference standard: final diagnosis by POC

POC was performed in 32 of 34 patients (94.1%). BA was confirmed on POC in 25 patients (73.5% of the total cohort; 78.1% of those who underwent POC). Non-BA cholestasis was confirmed in nine patients (26.5%). The two patients who did not undergo POC demonstrated clear biliary tracer excretion on HIDA scintigraphy and were classified as non-BA. Cystic BA was identified in two patients (5.9%).

Biochemical findings

The mean serum total bilirubin was 12.36 ± 5.63 mg/dL (BA subgroup: 11.58 mg/dL) and mean direct bilirubin was 6.74 ± 2.50 mg/dL (BA subgroup: 6.47 mg/dL). The mean serum GGT was 372.30 ± 340.58 IU/L (BA subgroup: 365.28 IU/L). Elevated GGT was not significantly associated with the diagnosis of BA on liver biopsy (p = 0.085). TORCH serology revealed CMV IgM positivity in three patients (8.8%) and CMV IgG positivity in 14 (41.2%). Rubella IgG was positive in two patients (5.9%).

Index test 1: HSF on color Doppler USG (primary index test)

Coarse heterogeneous hepatic echotexture was identified in eight patients (23.5%). The gallbladder was visualized in 29 patients (85.3%) and the common bile duct in six (17.6%). The triangular cord sign was detected in six patients (17.6%). Gallbladder visualization was not significantly associated with the liver biopsy diagnosis of BA (p = 0.559).

HSF was detected in 8 of 34 patients (23.5%). All eight patients in whom HSF was detected were subsequently confirmed to have BA on POC (Table 1). No patient without BA had detectable HSF. Conversely, 17 of 25 BA patients (68%) had no detectable HSF, representing a high false-negative rate.

**Table 1:** Diagnostic accuracy of HSF on color Doppler ultrasonography (primary index test) for the detection of biliary atresia. Reference standard: POC. Values in parentheses for sensitivity and specificity represent 95% CI. TP: true positive; FP: false positive; FN: false negative; TN: true negative; PPV: positive predictive value; NPV: negative predictive value; CI: confidence interval (Wilson score method); BA: biliary atresia; POC: per-operative cholangiogram; HSF: hepatic subcapsular flow

HSF on Color Doppler	POC: BA (n=25)	POC: Non-BA (n=9)
Present	8 (TP)	0 (FP)
Absent	17 (FN)	9 (TN)
Sensitivity	32.0%	14.9-53.5%
Specificity	100%	66.4-100%
PPV	100%	-
NPV	34.6%	-
Overall Accuracy	50.0%	-

The diagnostic accuracy of HSF as the primary index test for BA was: sensitivity 32.0% (95% CI: 14.9-53.5%), specificity 100% (95% CI: 66.4-100%), PPV 100%, NPV 34.6%, and overall accuracy 50.0%. These findings indicate that HSF, when present, is highly specific for BA in this cohort; however, its low sensitivity substantially limits its utility as an early or stand-alone diagnostic marker. No statistically significant association was found between HSF detection and liver biopsy diagnosis of BA (p = 0.397).

Index test 2: hepatobiliary scintigraphy

All 34 enrolled patients underwent HIDA scintigraphy. Definite biliary excretion into the intestine was demonstrated in only two patients (5.9%), both of whom were subsequently classified as non-BA. The remaining 32 patients demonstrated a non-excretion/obstructive pattern on scintigraphy and underwent POC. Among these, 25 were confirmed to have BA, and seven were diagnosed as non-BA. Using a non-excretion/obstructive scintigraphic pattern as a positive test for BA, HIDA scintigraphy demonstrated a sensitivity of 100% (95% CI: 86.3%-100%), specificity of 22.2% (95% CI: 2.8%-60.0%), PPV of 78.1%, NPV of 100%, and overall diagnostic accuracy of 79.4% (Table 2).

**Table 2:** Diagnostic accuracy of hepatobiliary scintigraphy (HIDA scan) as a comparator index test for the detection of biliary atresia. Reference standard: POC. All 34 patients underwent HIDA scintigraphy. Definite biliary excretion into the intestine was observed in only two patients, both classified as non-biliary atresia. The remaining 32 patients demonstrated a non-excretion/obstructive pattern, of whom 25 were confirmed as biliary atresia and seven as non-biliary atresia by POC. TP: true positive; FP: false positive; FN: false negative; TN: true negative; POC: per-operative cholangiogram; HIDA: hepatobiliary iminodiacetic acid; PPV: positive predictive value; NPV: negative predictive value; BA: biliary atresia

HIDA Scan	POC: BA (n = 25)	POC: Non-BA (n = 9)
Non-excretion / obstructive pattern (positive for BA)	25 (TP)	7 (FP)
Definite biliary excretion into bowel (negative for BA)	0 (FN)	2 (TN)
Sensitivity	100%	86.3-100%
Specificity	22.2%	2.8-60.0%
PPV	78.1%	-
NPV	100%	-
Overall Accuracy	79.4%	-

Index test 3: percutaneous liver biopsy

The mean Chen histopathological score was 9.08 in the BA group (n = 25) and 8.78 in the non-BA group (n = 9) (Table 3). There was no statistically significant difference between groups (independent t-test, p = 0.207), demonstrating the considerable histological overlap between BA and non-BA cholestasis in this cohort. 

**Table 3:** Comparison of mean Chen histopathological liver biopsy scores between the biliary atresia (BA) and non-BA groups, with per-operative cholangiogram (POC) as the reference standard. "*" Independent samples t-test; not statistically significant (p = 0.207). t-value = 1.28 (back-calculated from reported p-value; df = 32). Reference standard: POC. Chen scores are available for 22 of 25 BA patients and 9 of 9 non-BA patients. "-" indicates that the t-value and p-value apply to the overall group comparison, not to individual groups separately.

Group	n	Mean Chen Score (±SD)	t-value	p-value
Biliary Atresia (BA)	25	9.08 ± 3.18	1.28	0.207*
Non-BA Cholestasis	9	8.78 ± 2.52	-	-

Using a Chen score ≥8 as the threshold for BA suspicion, liver biopsy correctly identified all 25 BA patients (sensitivity 100%; 95% CI: 86.3-100%). However, seven of nine non-BA patients also scored ≥8, yielding a specificity of 22.2% (95% CI: 2.8-60.0%), PPV of 78.1%, NPV of 100%, and overall accuracy of 79.4% (Table 4). A statistically significant correlation was observed between a liver biopsy result suspicious for BA (Chen score ≥8) and confirmation of BA on POC (p = 0.014), supporting the role of liver biopsy as a high-sensitivity gatekeeper to POC, albeit at the cost of specificity. 

**Table 4:** Diagnostic accuracy of percutaneous liver biopsy using Chen score ≥8 as threshold (comparator index test) for the detection of BA. Reference standard: POC. Low specificity reflects the protocol's intent to maximize sensitivity and avoid missed biliary atresia diagnoses. Values in parentheses for sensitivity and specificity represent 95% CI (Wilson score method). TP: true positive; FP: false positive; FN: false negative; TN: true negative; POC: per-operative cholangiogram; PPV: positive predictive value; NPV: negative predictive value; BA: biliary atresia

Liver Biopsy (Chen Score)	POC: BA (n = 25)	POC: Non-BA (n = 9)
≥8 (BA suspected)	25 (TP)	7 (FP)
<8 (BA not suspected)	0 (FN)	2 (TN)
Sensitivity	100%	86.3-100%
Specificity	22.2%	2.8-60.0%
PPV	78.1%	-
NPV	100%	-
Overall Accuracy	79.4%	-

Correlation analysis

The correlations between liver biopsy findings (Chen score) and individual diagnostic parameters or the final POC diagnosis are summarized in Table 5. HSF (p = 0.397), gallbladder visualization (p = 0.559), and serum GGT (p = 0.085) were not significantly correlated with liver biopsy suspicion of BA. The POC diagnosis was significantly correlated with liver biopsy suspicion of BA (p = 0.014).

**Table 5:** Correlation of liver biopsy (Chen score) findings with POC diagnosis and other diagnostic parameters (Fisher's exact test / chi-square). NS = not significant (p ≥ 0.05). Chi-square (χ²) values shown for chi-square tests; Fisher’s exact test used where cell counts < 5. All p-values are two-tailed. Statistical tests: chi-square (POC and GGT); Fisher’s exact (HSF, gallbladder visualization). Liver biopsy result used as the dependent variable. GGT: gamma-glutamyl transpeptidase; POC: per-operative cholangiogram; HSF: hepatic subcapsular flow

Diagnostic Parameter	Test Statistic Value	p-value	Significance
Per-operative cholangiogram (POC) diagnosis	χ² = 5.903	0.014	Significant
Hepatic subcapsular flow on color Doppler	Fisher's exact	0.397	NS
Gallbladder visualization on ultrasonography	Fisher's exact	0.559	NS
Serum GGT level	χ² = 2.993	0.085	NS

Post-operative outcomes

Among 25 patients with BA who underwent Kasai portoenterostomy, jaundice clearance was achieved in eight (32%) at the last follow-up. Five patients had persistent jaundice at one month post-operatively, and two at six months. Three deaths (8.8% of the BA cohort) occurred during post-operative follow-up. Both patients with cystic BA achieved post-operative jaundice clearance. One patient with co-existent CMV IgM infection received adjuvant valganciclovir and became jaundice-free but experienced recurrent episodes of cholangitis.

## Discussion

This cross-sectional diagnostic accuracy study evaluated the performance of HSF on color Doppler USG, hepatobiliary scintigraphy (HIDA scan), and liver biopsy against POC as the reference standard for the diagnosis of BA in infants with NC. The principal findings were that HSF demonstrated high specificity (100%) but low sensitivity (32.0%) for BA, with an overall diagnostic accuracy of 50.0%, thereby limiting its value as a stand-alone diagnostic test. Liver biopsy demonstrated high sensitivity (100%) and overall accuracy (79.4%) within the diagnostic pathway used in this study. However, substantial overlap in histopathological findings between BA and non-BA cholestasis limited its discriminatory performance when used in isolation. HIDA scintigraphy demonstrated high sensitivity but low specificity, as most non-BA patients also showed an obstructive/non-excretory pattern.

The most important observation in this study is the substantially lower sensitivity of HSF compared with previously published studies. Lee et al. reported 100% sensitivity and 86% specificity, while El-Guindi et al. demonstrated sensitivity and specificity exceeding 96% [7,8]. In contrast, HSF was detected in only 8 of 25 patients with BA in the present cohort, corresponding to a sensitivity of 32.0% and an overall accuracy of 50.0%. A meta-analysis by Sun et al. reported pooled sensitivity and specificity values of 77.5% and 79.2%, respectively, but it also demonstrated considerable heterogeneity among included studies [9]. Several factors may explain the lower sensitivity observed in our cohort. First, the mean age at presentation was 3.09 months, which is beyond the optimal diagnostic and surgical window for BA. Delayed presentation may alter Doppler detectability, as progressive fibrosis and cirrhosis can alter hepatic arterial flow dynamics. Second, HSF assessment is inherently operator-dependent and was performed by multiple radiologists with varying levels of expertise in pediatric hepatobiliary imaging. Third, standardized pediatric criteria or quantitative thresholds for abnormal HSF have not yet been established, limiting reproducibility across centers. Together, these factors likely contributed to the reduced sensitivity observed in this study and highlight the challenges of translating HSF into routine clinical practice.

Despite its low sensitivity, HSF demonstrated 100% specificity in our cohort, as every patient with detectable HSF was confirmed to have BA on POC. This finding suggests that HSF, when confidently identified, may serve as a useful adjunctive confirmatory sign that can accelerate surgical referral. However, the absence of HSF cannot exclude BA and should not delay further diagnostic evaluation. Our findings therefore support HSF as a complementary imaging marker rather than an independent diagnostic test.

The diagnostic performance of HIDA scintigraphy observed in this study reinforces its established role as a highly sensitive but poorly specific investigation for BA in infants with NC. The low specificity observed in our cohort indicates that an obstructive or non-excretory scintigraphic pattern is not unique to BA and may also be seen in other cholestatic disorders with significant hepatocellular dysfunction. Similar limitations of HIDA scintigraphy have been reported in previous studies and systematic reviews [1,5,12]. The NASPGHAN/ESPGHAN guidelines likewise recommend that HIDA scintigraphy should not be used as a standalone diagnostic test for BA because absent biliary excretion can occur in both obstructive and severe non-obstructive cholestatic conditions [1]. The reduced specificity in our cohort was likely influenced by the late presentation and advanced cholestatic disease burden of the study population, which may impair biliary excretion irrespective of the underlying etiology.

Liver biopsy demonstrated high sensitivity (100%) within the diagnostic pathway used in this study, with an overall accuracy of 79.4%, findings that are broadly consistent with previous reports supporting its role in the evaluation of BA [12]. However, its specificity was low (22.2%), reflecting substantial histopathological overlap between BA and other causes of NC. This overlap was evident in our cohort, where the mean Chen scores did not differ significantly between BA and non-BA patients (9.08 vs. 8.78; p = 0.207). Although a Chen score ≥8 was significantly associated with POC-confirmed BA (p = 0.014), this finding should be interpreted cautiously because most non-BA patients (7/9) also exceeded this threshold. Taken together, these results suggest that while the Chen scoring system was useful for identifying patients who required further definitive evaluation, its ability to discriminate reliably between BA and non-BA cholestasis was limited in our cohort. The limited discriminatory performance may reflect the relatively late age at presentation, advanced cholestatic liver injury and fibrosis in both groups, and the small number of non-BA patients. These findings underscore the limitations of relying solely on composite histopathological scores for diagnosis. Individual histological features such as bile duct proliferation and portal fibrosis, which have been reported as more specific indicators of BA [12], may provide greater diagnostic value and warrant focused evaluation in future studies.

The postoperative jaundice clearance rate following Kasai portoenterostomy was 32%, which is lower than rates reported by high-volume centers [3]. This likely reflects delayed presentation and referral, as the mean age at diagnosis in our cohort exceeded the recommended surgical window of 60 days. Late presentation and delayed diagnosis remain a major challenge in resource-limited settings and contribute significantly to poorer native liver survival outcomes. Improving awareness of early evaluation of persistent neonatal jaundice and of timely referral pathways may therefore have a greater clinical impact than refining individual diagnostic tests alone.

This study has several important limitations. First, the small number of non-BA patients (n = 9) resulted in wide confidence intervals around specificity estimates, limiting their precision and clinical interpretability. Consequently, the observed specificity values should be regarded as preliminary estimates requiring validation in larger cohorts. Recruitment was further affected by the COVID-19 pandemic, which restricted enrollment during the study period and contributed to the limited sample size.

Second, the relatively late age at presentation in our cohort (mean 3.09 ± 1.46 months) introduced potential selection bias toward patients with more advanced biliary fibrosis and cirrhosis. As HSF is hypothesized to reflect hyperplastic arterial changes associated with progressive fibrosis, disease stage may have influenced its detectability in our cohort. However, the relationship between disease duration, severity of fibrosis, and HSF detection remains uncertain and could not be evaluated in the present study. Furthermore, the diagnostic performance of HSF in earlier-presenting infants - the population in whom an early non-invasive marker would be of greatest clinical value - cannot be inferred from our data.

Third, HSF assessment was performed by multiple radiologists with varying levels of experience in pediatric hepatobiliary imaging, and no standardized acquisition protocol or validated pediatric threshold criteria were available. Because inter-observer agreement was not prospectively assessed, the extent to which operator variability influenced HSF detection could not be quantified. These factors may have contributed substantially to the low sensitivity observed and highlight the need for standardized imaging protocols and formal inter-observer reliability assessments in future studies.

Fourth, the limited sample size reduced statistical power and increased uncertainty around diagnostic accuracy estimates. Consequently, the study may have been underpowered to detect clinically meaningful differences between diagnostic modalities or to provide precise estimates of specificity. This limitation is particularly relevant to the non-significant difference in Chen scores between BA and non-BA patients, which should be interpreted cautiously given the small sample size and limited number of non-BA cases.

Finally, this was a single-center study conducted at a tertiary referral institution, which may limit the generalizability of the findings to other healthcare settings, particularly centers with different referral patterns, patient populations, imaging expertise, and diagnostic workflows.

Despite these limitations, the study provides clinically relevant real-world data from a tertiary pediatric surgical center and directly compares HSF, HIDA scintigraphy, and liver biopsy against POC within the same patient cohort. The findings reinforce the importance of multimodal evaluation in NC and highlight the limitations of relying on any single diagnostic investigation for BA.

In summary, HSF on color Doppler USG demonstrated excellent specificity but limited sensitivity for BA in this cohort, supporting its role as an adjunctive rather than definitive diagnostic marker. Liver biopsy provided useful complementary diagnostic information, although its discriminatory performance was limited by substantial histopathological overlap between BA and non-BA cholestasis, while HIDA scintigraphy remained highly sensitive but poorly specific. Larger multicenter prospective studies with standardized Doppler protocols and earlier patient enrollment are required to better define the clinical utility of HSF in the diagnostic evaluation of BA.

## Conclusions

HSF on color Doppler USG demonstrated high specificity but low sensitivity for the diagnosis of BA in infants with NC, limiting its role as a stand-alone diagnostic test. Liver biopsy demonstrated high sensitivity within the diagnostic pathway studied and provided useful complementary information when interpreted alongside clinical, imaging, and operative findings. HIDA scintigraphy demonstrated high sensitivity but low specificity in differentiating BA from non-BA cholestatic disorders. These findings support the continued use of a multimodal diagnostic approach for NC. Larger multicenter studies with standardized Doppler protocols and earlier patient enrollment are required to better define the clinical utility of HSF in BA diagnosis.
